# Supplementary material for: Soluble and Exosome-Bound α-Galactosylceramide Mediate Preferential Proliferation of Educated NK Cells with Increased Anti-Tumor Capacity
Source: Cancers (Basel). 2021 Jan 15;13(2):298. doi: 10.3390/cancers13020298 (PMC7830699; doi:10.3390/cancers13020298)
Supplement: Supplementary file 1 [file cancers-13-00298-s001.pdf]

cancers

MDPI

Supplementary Materials: Soluble and Exosome-Bound α-Galactosylceramide Mediate Preferential Proliferation of Educated NK Cells with Increased Anti-Tumor Capacity

Arnika K. Wagner, Ulf Gehrmann, Stefanie Hiltbrunner, Valentina Carannante, Thuy T. Luu, Tanja I. Näslund, Hanna Brauner, Nadir Kadri, Klas Kärre and Susanne Gabrielsson

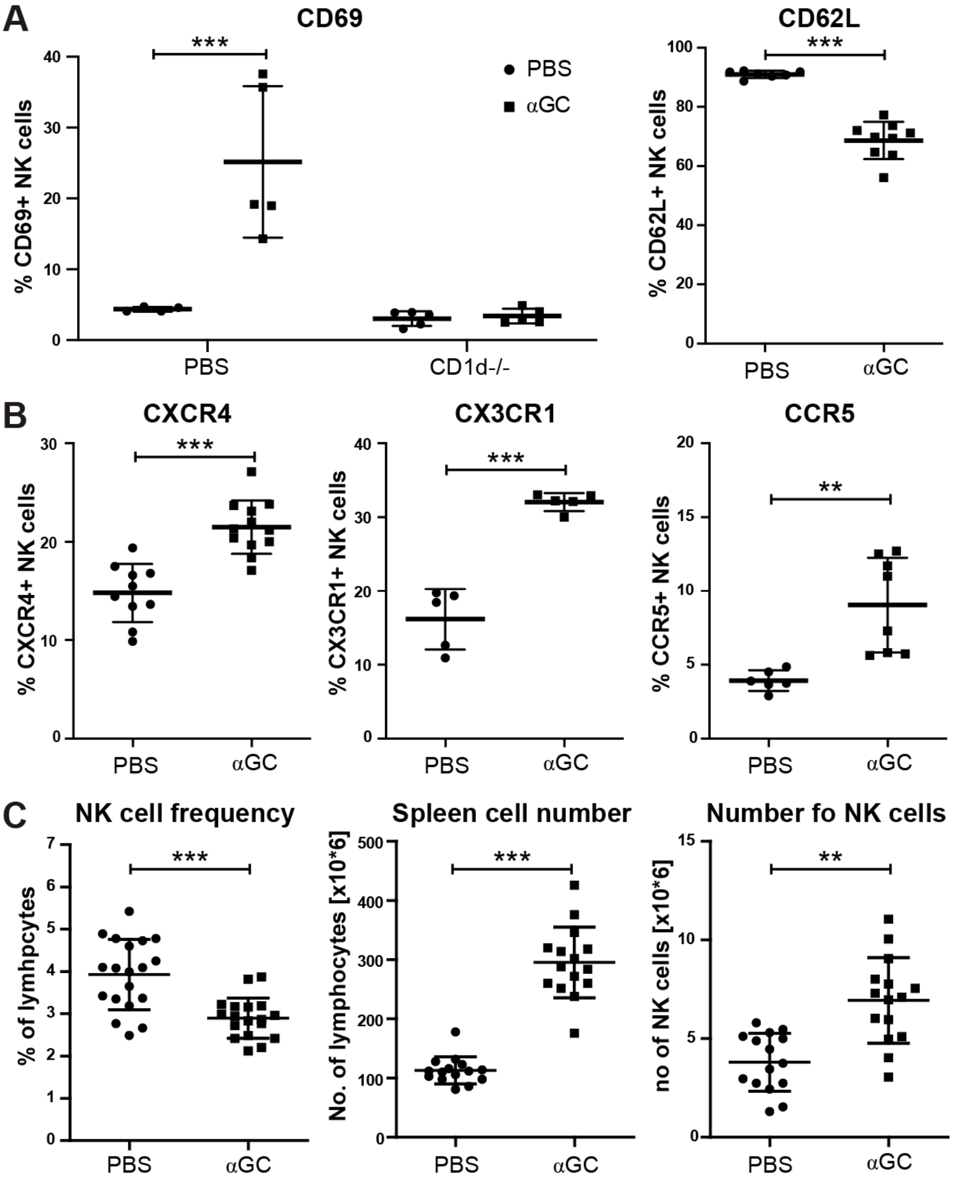

Figure S1. iNKT cell-dependent activation of NK cells by αGC in vivo. C57Bl/6 or CD1d$^{-/-}$ mice were injected i.v. with PBS or 200ng soluble αGC. (**a**) Frequency of activation markers was measured on day 2 (CD69^{+} NK cells) or day 7 (CD62L^{+} NK cells) after treatment. (**b**) Frequency of chemokine receptors CXCR4, CX3CR1 and CCR5 was assessed at day 7 post-treatment. (**c**) NK cell frequency, spleen cell number and number of NK cells was determined at day 7 after treatment with αGC. Data are pooled from 2–4 independent experiments and show 4-9 mice per group (**a**), 5–12 mice per group (**b**) and 15–19 mice per group (**c**). Error bars denote SD. Significant differences were calculated with

two-way ANOVA with Tukey's correction for multiple testing (**a, CD69**) or unpaired two-tailed t test for all other subfigures and are denoted ** $p < 0.01$, *** $p < 0.001$.

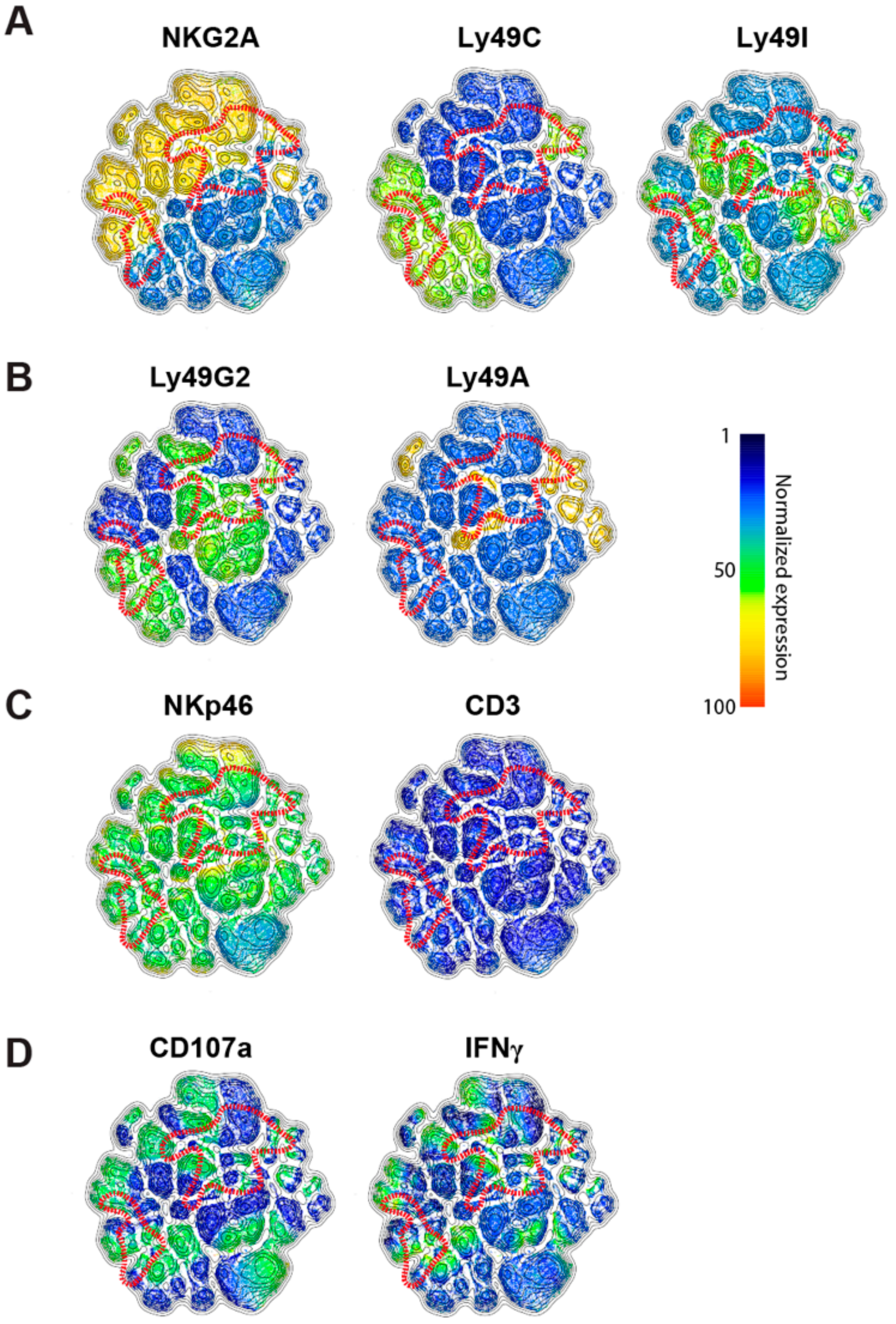

Figure S2. tSNE analysis of NK cell receptors. Flow cytometry data of mice of four groups (B6, B6+αGC, CD1d-/-, CD1d-/-+αGC) was analyzed with t-distributed stochastic neighbor embedding algorithm as previously described[39]. tSNE plots of individual educating inhibitory receptors (**a**), non-educating inhibitory receptors (**b**), NK cell defining markers (**c**) and functional markers (**d**). Gates were determined manually on areas with 10% increase of cells on tSNE plots of Fig. 2F and

subsequently applied to all remaining tSNE plots. Data is pooled from 3 independent experiments with 6 mice per group.

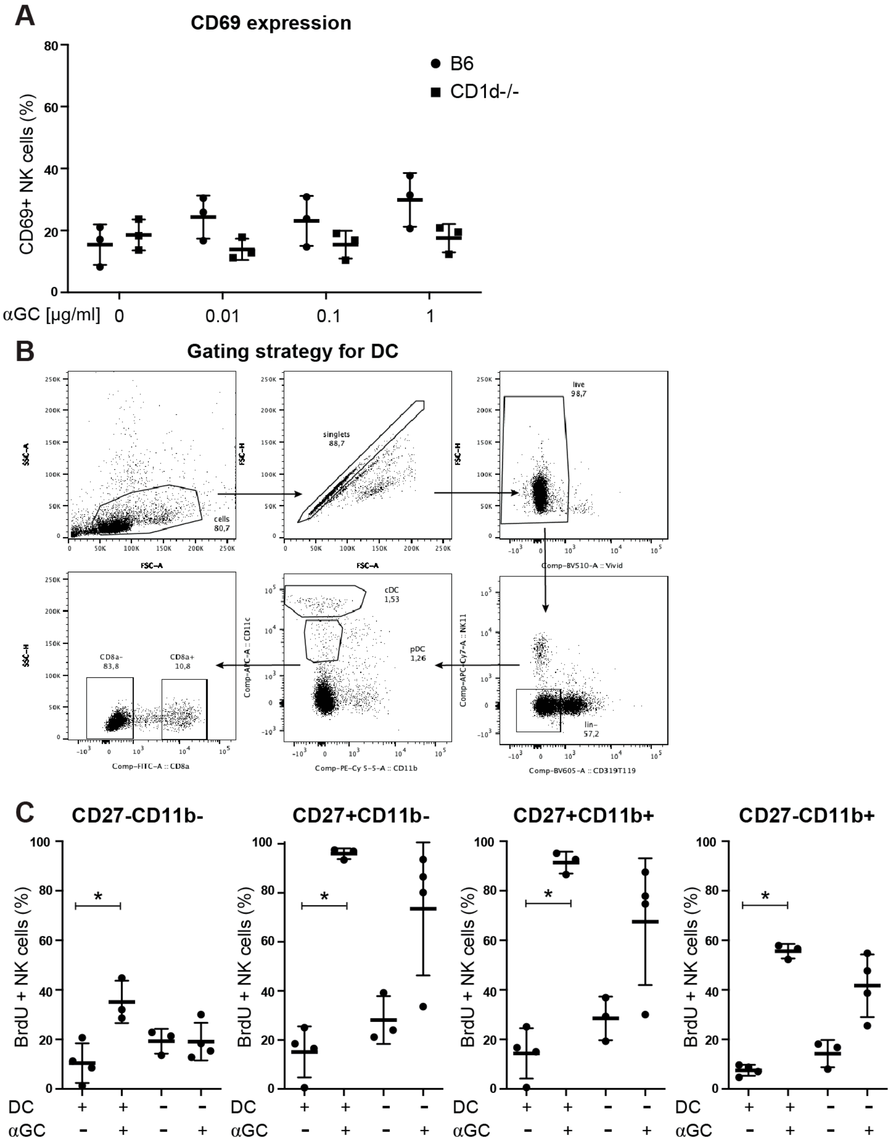

Figure S3. DCs are necessary for NK cell activation. (**a**) Spleen cells of B6 and CD1d$^{-/-}$ were isolated and cultured *in vitro* in the presence of absence of αGC (00.1, 0.1, 1 µg/ml). Total spleen cells were cultured and stimulated and CD69 expression measured on NK cells after 24h. (**b**) Gating strategy for CD8α^{+} cDC1 cells. (**c**) CD11c.DOG mice were treated with PBS (DC+) or DTR (DC−) for 7 d and received PBS or 200ng of αGC and were fed BrdU via the drinking water from day 3. NK cells were stained at day 7 and maturation markers CD27 and CD11b and KLRG1 expression was assessed on these maturational subsets. Shown are data from 3 mice per group from one experiment (**a**) or one experiment with 3-5 mice per group (**b**). Two-way ANOVA with Tukey's multiple comparison test (**a**) or Kruskal_wallis test with Dunn's correction for multiple testing (**c**) was used to determine statistical significance. Error bars indicate SD. * $p < 0.05$.

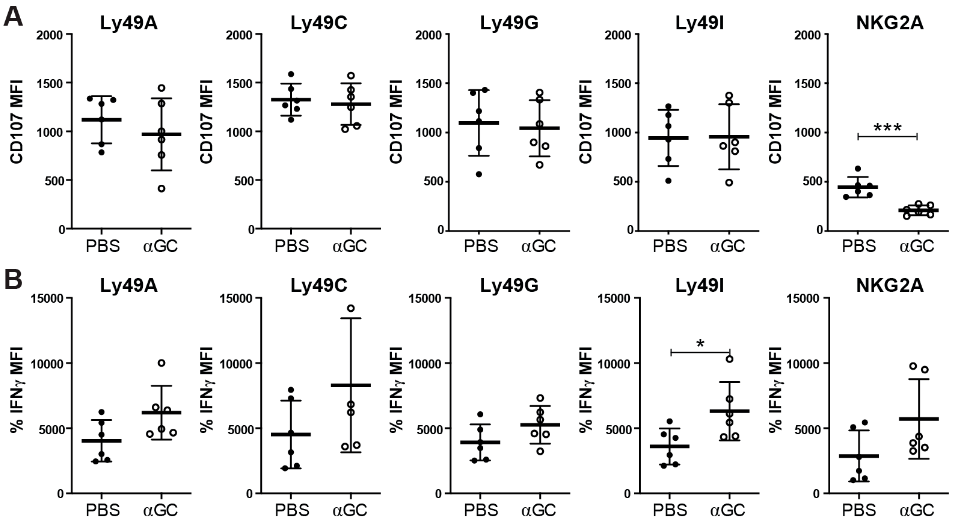

Figure S4. PMA/ionomycin-stimulated NK cells show minimal changes in functional response. C57Bl/6 mice were injected i.v., with PBS or 200 ng soluble *α*GC. 3 d after in vivo stimulation, degranulation (by CD107 positivity) and cytokine production capacity (IFNγ production) were assessed as for Figure 3. (**a**) CD107 and (**b**) IFNγ production of C57Bl/6 NK cell subsets based on the inhibitory receptors (Ly49G2 vs. Ly49A, Ly49C vs. Ly49I, NKG2A) stimulated with PMA/ionomycin. Data is pooled from 3 independent experiments with 6 mice per group. Unpaired, two-tailed t-test was used to determine statistical significance. Error bars indicate * $p < 0.05$.

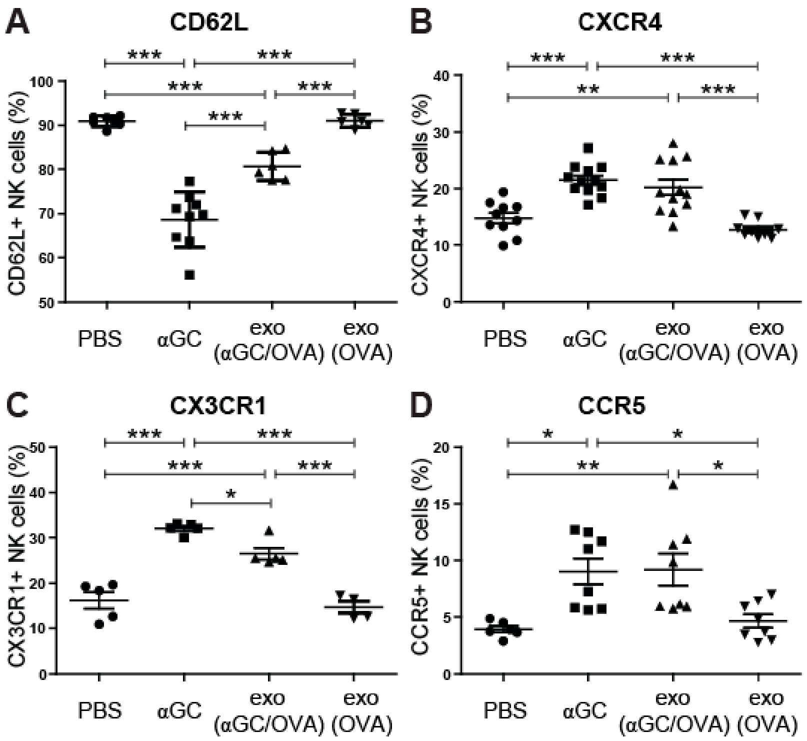

Figure 5. Exosomal *α*GC has comparable effects on NK cell activation, proliferation and missing self-function. C57Bl/6 mice were injected i.v. with PBS, 40 µg Exo(*α*GC-OVA) or Exo(*α*GC) or 200 ng soluble *α*GC (equivalent to the amount on 40 µg Exo(*α*GC-OVA)/Exo(*α*GC) [32]. Expression of surface markers was measured by flow cytometry on d7. Expression of adhesion molecule (**a**) CD62L, and chemokine receptors (**b**) CXCR4, (**c**) CX3CR1, and (**d**) CCR5 are shown on total NK cells. Data are pooled from 2–4 independent experiments with 4–12 mice per group. Significant differences were calculated with one-way ANOVA with Tukey's correction for multiple testing and are denoted * $p < 0.05$, ** $p < 0.01$, *** $p < 0.001$.

© 2021 by the authors. Licensee MDPI, Basel, Switzerland. This article is an open access article distributed under the terms and conditions of the Creative Commons Attribution (CC BY) license (http://creativecommons.org/licenses/by/4.0/).
